# Seroprevalence and demographic factors associated with hepatitis B, hepatitis C and HIV infection from a hospital emergency department testing programme, London, United Kingdom, 2015 to 2016

**DOI:** 10.2807/1560-7917.ES.2019.24.27.1800377

**Published:** 2019-07-04

**Authors:** Nick Bundle, Sooria Balasegaram, Sarah Parry, Sadna Ullah, Ross J Harris, Karim Ahmad, Graham R Foster, Cheuk YW Tong, Chloe Orkin

**Affiliations:** 1 United Kingdom Field Epidemiology Training Programme, Public Health England, London, United Kingdom; 2 Field Epidemiology Services, National Infection Service, Public Health England, London, United Kingdom; 3 Queen Mary University of London, London, United Kingdom; 4 Statistics, Modelling and Economics Department, National Infection Service, Public Health England, London, United Kingdom; 5 Barts Health NHS Trust, Emergency Department, Royal London Hospital, London, United Kingdom; 6 Blizard Institute, Queen Mary University, London, United Kingdom; 7 Barts Health NHS Trust, Virology department, Royal London Hospital, London, United Kingdom

**Keywords:** hepatitis B, hepatitis C, human immunodeficiency virus, HIV, blood-borne virus testing, emergency department, seroprevalence

## Abstract

**Background:**

Progress towards HIV, hepatitis B virus (HBV) and hepatitis C virus (HCV) elimination requires local prevalence estimates and linkage to care (LTC) of undiagnosed or disengaged cases.

**Aim:**

We aimed to estimate seroprevalence, factors associated with positive blood-borne virus (BBV) serology and numbers needed to screen (NNS) to detect a new BBV diagnosis and achieve full LTC from emergency department (ED) BBV testing.

**Methods:**

During a 9-month programme in an ED in east London, England, testing was offered to adult attendees having a full blood count (FBC). We estimated factors associated with positive BBV serology using logistic regression and NNS as the inverse of seroprevalence. Estimates were weighted to the age, sex and ethnicity of the FBC population.

**Results:**

Of 6,211 FBC patients tested, 217 (3.5%) were positive for at least one BBV. Weighted BBV seroprevalence was 4.2% (95% confidence interval (CI): 3.6–4.9). Adjusted odds ratios (aOR) of positive BBV serology were elevated among patients that were: male (aOR: 2.7; 95% CI: 1.9–3.9), 40–59 years old (aOR: 1.9; 95% CI: 1.4–2.7), of Black British/Black other ethnicity (aOR: 1.8; 95% CI: 1.2–2.8) or had no fixed address (aOR: 2.9; 95% CI: 1.5–5.5). NNS to detect a new BBV diagnosis was 154 (95% CI: 103–233) and 135 (95% CI: 93–200) to achieve LTC.

**Conclusions:**

The low NNS suggests routine BBV screening in EDs may be worthwhile. Those considering similar programmes should use our findings to inform their assessments of anticipated public health benefits.

## Introduction

Global strategies to eliminate the blood-borne viruses (BBV) hepatitis B virus (HBV), hepatitis C virus (HCV) and HIV as a public health threat by 2030 [1,2] require estimates of local prevalence in population subgroups [3], detection of undiagnosed infection, better linkage to care (LTC) and treatment of infected individuals [4-7]. In the United Kingdom (UK), the BBV burden occurs disproportionately in London, a demographically diverse city that is home to a third of all people diagnosed with HCV in England [8] and 40% of those living with HIV in the UK [9]. The city also has a rate of positive hepatitis B (HBV) tests (1.1%) from antenatal screening that is more than double the English average [10]. London-wide modelled prevalence estimates for people 15–59 years old exist for HCV (1.2%; 95% credible interval (CrI): 0.9–1.8) [11] and diagnosed HIV (0.57%; 95% confidence interval (CI): 0.56–0.58) [12], but are not yet available for HBV. Rates of infection vary considerably according to demographic and lifestyle risk factors [4,8,10,13,14] and 11% of HIV, 40% of HCV and an unknown number of HBV infections in London [8,9] are thought to be undiagnosed.

Current UK guidelines recommend routine screening for HIV in a range of medical settings, including hospital emergency departments (EDs) in areas of high prevalence (> 0.2%) [15,16]. Similar recommendations for HBV and HCV do not exist, with testing usually occurring in specialist addiction units, prisons and sexual health centres. Short-duration screening campaigns in urban EDs in the UK have detected high BBV seroprevalence of between 1.2–2.6% for HCV [17,18], 0.7% for HBV [17] and 0.2–0.8% for HIV [17,19]. ED testing has shown to be acceptable to staff and patients [19] and does not adversely affect length of stay when offered to patients having routine blood tests [20]. Testing for BBVs in EDs is recommended in the United States (US) as part of birth-cohort HCV testing [21,22] and universal screening programmes have been carried out in Europe [23-25], but until now no prospective combined BBV testing programmes of a duration longer than 1 week have been reported in the UK.

As an extension of a previous week-long campaign named Going Viral [17], we carried out a 9-month prospective BBV testing programme with LTC in the ED of an east London hospital to assess the feasibility of routinely providing ED-based opt-out testing. The programme had complementary clinical and epidemiological components, the former being the focus of a separate paper [26]. Here we describe the epidemiological component, which had the following objectives: (i) to identify factors associated with the uptake of BBV testing and positive BBV serology, (ii) to estimate BBV seroprevalence and numbers needed to screen (NNS) to detect a new diagnosis and fully link a case to care and (iii) to describe the diagnostic status of BBV cases and the LTC outcomes achieved in the first 6 months after the end of testing.

## Methods

### Programme overview

Between 20 November 2015 and 7 August 2016, BBV opt-out testing was offered to adult ED attendees who had the capacity to consent verbally and had a full blood count (FBC) as part of routine care. An additional blood sample was taken and tested for HIV antigen/antibody, HBV surface antigen (HBsAg) and HCV antibody (HCV-Ab), with reactive HCV samples subsequently tested for HCV RNA. The specific laboratory methods used have been described previously [26]. LTC started as soon as the first cases were identified and continued after the programme as part of normal clinical follow-up.

### Case definitions

#### Infection-specific cases

HIV, HBV and HCV-RNA cases were defined as an ED attendee who had a FBC with a reactive result for HIV antigen/antibody, HBsAg or HCV RNA, respectively. An inclusion criterion of having been tested for at least two BBVs reflected the programme’s intention of testing patients for multiple infections, while accepting that patients could choose to opt out of specific tests.

#### BBV case

A BBV case was defined as an ED attendee who had a FBC and was tested for at least two BBVs, with a reactive result for HIV antigen/antibody and/or HBsAg and/or HCV RNA.

#### Diagnostic status of cases

All cases were assigned to one of five categories of diagnostic status based on information they provided when they were contacted by phone to notify them of their positive result, or from searches of hospital laboratory records (Table 1). While diagnostic status was obtained for all patients positive for HCV-Ab [26], it is only presented for HCV-RNA cases.

**Table 1:** Categories of diagnostic status of blood-borne virus cases, hospital emergency department testing programme, London, United Kingdom, 2015–2016

Category	Information obtained from the patient or through hospital laboratory records
Known-engaged	Patient had been previously diagnosed or had a previous positive test result on hospital laboratory records and was enrolled in healthcare to treat or manage the disease
Uncontactable	Patient could not be contacted to be notified of their diagnosis and had no previous test result on hospital records so diagnostic status remained unknown
New diagnosis	Patient informed us that their diagnosis was not previously known to them
Known-disengaged	Patient had been previously diagnosed or had a previous positive test result on hospital laboratory records, but was lost to follow-up from healthcare to treat or manage the disease
Known-unknown	Patient had a previous positive result in hospital records, but could not be contacted to determine whether they were engaged in healthcare

#### Cases requiring linkage to care

Cases requiring linkage to care (RLTC) were defined as living BBV cases whose diagnostic status was classified as new diagnosis, known-disengaged, known-unknown or uncontactable. Known-engaged BBV cases did not require LTC, but were included in seroprevalence estimates.

#### Linkage to care outcomes

The clinical team’s records of attempts to contact cases and the number of clinics/inpatient consultations attended by those RLTC were used to assign three sequential LTC outcomes: notified (informed of positive test result), partially linked to care (notified and attended one clinic or inpatient consultation) and fully linked to care (notified and attended either two clinics or one inpatient consultation and one clinic).

### Data collection and management

Datasets of ED attendees, FBC patients and BBV tests were extracted from the hospital IT system and medical staff recorded the follow-up of cases RLTC. Data were imported into Stata 14.1 (Stata Corp, College Station, Texas, US) for manipulation and analysis. We grouped variables related to patient demographics—sex, age, ethnicity and whether or not they had a fixed address (‘no fixed address’ being a proxy for homelessness)—into clinically and epidemiologically relevant categories and also created variables relating to ED arrival and waiting times, categorised as day/night, weekday/weekend and quartiles of time from arrival to assessment. We excluded duplicate records, patients < 18 years old, those missing a hospital number (a unique identifier to link a patient between datasets) or those in the BBV tests dataset that did not have a corresponding entry in the FBC or ED datasets. For patients with multiple tests or visits to the ED, we retained the most complete test record, i.e. the one that showed a change in infection status or the earliest record.

### Statistical analysis

#### Factors associated with uptake of BBV testing

We described the distribution of patients attending the ED, having a FBC and being tested for at least two BBVs across patient demographic and ED process variables. To estimate factors associated with uptake of BBV testing we identified variables associated with testing among all patients having a FBC using chi-squared tests. Sex, age, time of arrival in the ED and if they had no fixed address were subsequently included as independent variables in a logistic regression model, with BBV testing as the dependent variable to obtain adjusted odds ratios (aOR) for the association between each variable and BBV testing. We excluded patients whose records were missing their sex or waiting time, or whose age was unclassified, and estimated p values using a likelihood ratio test (LRT).

#### BBV seroprevalence and demographic factors associated with positive BBV serology

Crude seroprevalence was estimated separately for all cases, new diagnoses, cases RLTC and fully linked cases, as the proportion among the total tested for each. In addition to BBV cases and the three infection-specific cases, estimates of HCV-Ab seroprevalence are presented to facilitate comparison with published studies, since HCV RNA is relatively rarely reported. As the distribution of demographic variables varied between those tested and those not tested for BBVs, we adjusted for non-participation using survey weights based on the inverse probability of a patient who had a FBC being in each combination of age, sex and ethnicity, once those patients with unclassified age and sex were excluded. Survey weights were applied in estimations of both seroprevalence and demographic factors associated with positive BBV serology, described below. The intention of the weighted analyses were to derive adjusted estimates applicable to the whole population of patients requiring a FBC and not just those tested for BBVs.

To identify demographic factors associated with positive BBV serology we applied the general modelling process described above for BBV testing uptake, with some small differences: only demographic variables were considered, positive serology was used as the dependent variable in the regression model and, as survey weights were applied to the model, p values had to be estimated from a Wald test rather than a LRT. A model was fitted for all BBVs, as well as three separate infection-specific models for HCV RNA, HBV and HIV. Sex, age and ethnicity were included as independent variables in all four models and having no fixed address was included only for BBV, HBV and HCV RNA. We used the robust (Huber-White) standard error when estimating parameters and their variances in the weighted logistic model and compared these with estimates obtained from an unweighted model to confirm that weighting did not introduce model instability or materially change our conclusions. To avoid potential inflation of estimates that might arise due to data sparsity [27], we grouped cases into fewer age categories than reported elsewhere for this programme [26]. For the same reason, we also considered collapsing the ‘White British’ and ‘White other’ ethnic categories to reduce uncertainty in the estimates for ethnicity in HBV arising from small counts in the reference category, but decided against this on the grounds that maintaining the distinction between these groups was epidemiologically important and that our checks concluded no additional instability had been introduced by doing so.

#### Linkage to care outcomes 6 months after testing and numbers needed to screen

LTC was described according to the number and proportion of cases RLTC that had achieved the three sequential linkage outcomes by 30 January 2017. We calculated the NNS to identify one new diagnosis or to fully link a case to care by taking the inverse of the point estimate and 95% CI of adjusted prevalence among new diagnoses and fully linked cases, respectively [28].

### Ethical statement

The reason for the ethics committee’s decision that a formal ethics application was not required, as well as the attempts to contact all patients who tested positive to notify them of their results and invite them for clinical review with appropriate referral, have been described previously [26].

## Results

### Patient characteristics and factors associated with BBV testing uptake

After removing duplicates and exclusions (n = 31,427) there were 65,136 unique ED attendances by patients that were predominantly young (60% were aged 18–39 years and 14% were aged 60–89 years), of Asian British/Asian other (28%) or White British (25%) ethnicity and male (53%). The 24,981 (38%) who went on to have a FBC had no difference by sex and an older age distribution (44% aged 18–39 years and 25% aged 60–89 years). The median age (interquartile range (IQR)) of patients with a valid age recorded was 34 (IQR: 26–49) years old among ED attendees, 42 (IQR: 29–60) years old among those who had a FBC and 41 (IQR: 29–57) years old among those tested for BBVs; furthermore, 6,211 FBC patients were tested for two (n = 280) or three (n = 5,931) BBVs, giving a BBV testing uptake of 25% (Figure, Table 2).

**Figure:**
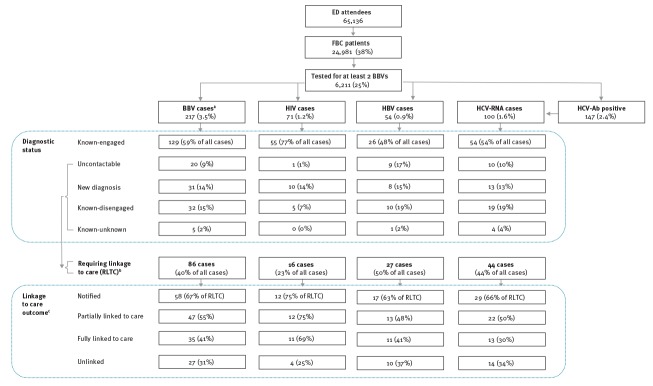
Flow of patients through the testing and linkage to care pathway, hospital emergency department testing programme, London, United Kingdom, 2015–2016

BBV: blood-borne virus; ED: emergency department; FBC: full blood count; HBV: hepatitis B virus; HCV: hepatitis C virus; RLTC: requiring linkage to care.

^a^ Any differences between the BBV totals and the infection-specific totals are due to co-infections (eight in total).

^b^ One new HBV diagnosis and two known-disengaged HCV-RNA cases died after their status was confirmed; therefore, they are not included in the RLTC total.

^c^ Percentages of cases achieving each linkage outcome are the proportions of all RLTC cases. As these outcomes are not mutually exclusive (e.g. to be partially linked one must also be notified) they can sum to more than 100%.

**Table 2:** Baseline characteristics and factors associated with testing for blood-borne viruses among patients having a full blood count, hospital emergency department testing programme, London, United Kingdom, 2015–2016 (n = 65,136)

Characteristics	ED attendees		FBC patients		Patients tested for at least 2 BBVs		
	n	%	n	%	n	%	aOR (95% CI)^a^
**Sex**							
Female	30,585	47	12,447	50	2,905	47	Ref
Male	34,543	53	12,532	50	3,306	53	1.2 (1.1–1.3)
**Age (years)**							
18–39	39,356	60	11,026	44	2,870	46	Ref
40–59	15,687	24	6,982	28	1,877	30	1.0 (0.94–1.1)
60–89	9,202	14	6,314	25	1,361	22	0.76 (0.70–0.82)
Unclassified^b^	891	1	659	3	103	2	NA
**Ethnicity**							
White British ethnicity	15,979	25	7,036	28	1,709	28	Ref
White other (incl. Irish)	11,092	17	3,078	12	819	13	1.1 (0.98–1.2)
Asian British/Asian other	17,953	28	6,721	27	1,749	28	1.1 (0.99–1.2)
Black British/Black other	5,268	8	2,233	9	603	10	1.1 (1.01–1.3)
Mixed or other	6,858	11	2,407	10	618	10	1.0 (0.93–1.2)
Ethnicity not recorded	7,986	12	3,506	14	713	11	0.78 (0.71–0.87)
**Residence**							
Fixed address	NA	NA	24,181	97	6,056	98	Ref
No fixed address	NA	NA	800	3	155	2	0.80 (0.65–0.98)
**Time of arrival in the ED**							
Daytime (08:00–19:59)	44,263	68	15,930	64	4,134	67	Ref
Night-time (20:00–07:59)	20,873	32	9,051	36	2,077	33	0.86 (0.81–0.91)
**Day of arrival in the ED**							
Weekday	47,790	73	18,507	74	4,566	74	NA
Weekend	17,346	27	6,474	26	1,645	26	NA
**Quartile of waiting time in the ED**							
Q1 (0–8 min)	16,341	25	7,879	32	1,881	30	NA
Q2 (9–19 min)	16,796	26	6,834	27	1,725	28	NA
Q3 (20–37 min)	15,724	24	5,232	21	1,312	21	NA
Q4 (38–805 min)	16,274	25	5,036	20	1,293	21	NA
**Total^c^**	**65,136**	**100**	**24,981**	**100**	**6,211**	**100**	**NA**

aOR: adjusted odds ratio; BBV: blood-borne virus; CI: confidence interval; ED: emergency department; FBC: full blood count; incl.: including; min: minutes; NA: not applicable; Q: quartile; Ref: reference category.

^a^ The aOR and 95% CI of having a BBV test was estimated using logistic regression. Likelihood ratio test p values: p < 0.0001 for sex, age and time of arrival and p = 0.0267 for no fixed address. Day of the week and waiting time were not included in the model, as they were not significantly associated with testing uptake in single variable analysis (p = 0.237 and p = 0.087, respectively).

^b^ Age group was coded as unclassified for patients with an invalid date of birth (n = 430) or whose age was recorded as > 89 years (n = 461 aged 90–111 years). Median (interquartile range) ages of patients with a valid age recorded were 34 (26–49) years old for ED attendees, 42 (29–60) years old for FBC patients and 41 (29–57) years old for BBV-tested patients.

^c^ Eight ED attendees with missing sex and one with a missing valid waiting time account for the differences between the overall total and the sum of their categories.

In the multivariable analysis, aOR of BBV testing were slightly elevated among FBC patients that were male (aOR: 1.2; 95% CI: 1.1–1.3) or of Black British/Black other ethnicity (aOR: 1.1; 95% CI: 1.0–1.3), compared to those who were female or White British, respectively. Patients ≥ 60 years old (aOR: 0.78; 95% CI: 0.71–0.87), with no fixed address (aOR: 0.80; 95% CI: 0.65–0.98), whose ethnicity was not recorded (aOR: 0.78; 95% CI: 0.71–0.87) or who arrived in the ED at night (aOR: 0.86; 95% CI: 0.81–0.91) had lower aOR of BBV testing than those who were aged 18–39 years, White British, had a fixed address or arrived in the day, respectively (Table 2).

### Seroprevalence and factors associated with positive BBV serology

There were 217 BBV cases identified among the 6,211 patients tested (3.5%), resulting in an estimated overall adjusted BBV seroprevalence of 4.2% (95% CI: 3.6–4.9) in FBC patients. Three cases were co-infected with HBV and HIV, and five with HCV and HIV; no HBV-HCV co-infections were detected. The adjusted seroprevalence was 1.6% (95% CI: 1.3–2.1) in the 86 cases RLTC and 0.7% (95% CI: 0.4–1.0) in the 31 new BBV diagnoses.

Elevated aOR of positive BBV serology were estimated in FBC patients that were male (aOR: 2.7; 95% CI: 1.9–3.9), aged 40–59 years (aOR: 1.9; 95% CI: 1.4–2.7), of Black British/Black other ethnicity (aOR: 1.8; 95% CI: 1.2–2.8) or who had no fixed address (aOR: 2.9; 95% CI: 1.5–5.5), compared to patients in the reference categories of female, aged 18–49 years, White British or of fixed address, respectively (Table 3).

**Table 3:** Seroprevalence of blood-borne viruses and demographic factors associated with positive BBV serology among patients having a full blood count, hospital emergency department testing programme, London, United Kingdom, 2015–2016 (n = 217)

Characteristic	Number tested	All cases			New diagnoses		RLTC	
		n	Prevalence % (95% CI)	aOR (95% CI)^a^	n	Prevalence % (95% CI)	n	Prevalence % (95% CI)
**Sex**								
Female	2,905	51	1.8 (1.3–4.0)	Ref	8	0.3 (0.1–0.6)	21	0.7 (0.5–1.1)
Male	3,306	166	5.0 (4.3–5.8)	2.7 (1.9–3.9)	23	0.7 (0.5–1.0)	65	2.0 (1.5–2.5)
**Age (years)**								
18–39	2,870	78	2.7 (2.2–3.4)	Ref	11	0.4 (0.2–0.7)	29	1.0 (0.7–1.5)
40–59	1,877	110	5.9 (4.9–7.0)	1.9 (1.4–2.7)	16	0.9 (0.5–1.4)	45	2.4 (1.8–3.2)
60–89	1,361	26	1.9 (1.3–2.8)	0.88 (0.53–1.5)	4	0.3 (0.1–0.8)	9	0.7 (0.3–1.3)
Unclassified	103	3	2.9 (0.9–8.6)	NA	0	0	3	2.9 (0.9–8.6)
**Ethnicity**								
White British	1,709	64	3.7 (2.9–4.8)	Ref	6	0.4 (0.2–0.8)	23	1.3 (0.9–2.0)
White other (incl. Irish)	819	43	5.3 (3.9–7.0)	1.5 (1.0–2.4)	10	1.2 (0.7–2.3)	24	2.9 (2.0–4.3)
Asian British/Asian	1,749	23	1.3 (0.9–2.0)	0.36 (0.22–0.60)	4	0.2 (0.1–0.6)	9	0.5 (0.3–1.0)
Black British/Black	603	42	7.0 (5.2–9.3)	1.8 (1.2–2.8)	5	0.8 (0.4–2.0)	10	1.7 (0.9–3.1)
Mixed or other	618	27	4.4 (3.0–6.3)	1.1 (0.69–1.9)	4	0.7 (0.2–1.7)	11	1.8 (1.0–3.2)
Ethnicity not recorded	713	18	2.5 (1.6–4.0)	0.55 (0.29–1.0)	2	0.3 (0.1–1.1)	9	1.3 (0.7–2.4)
**Residence**								
Fixed address	6,056	198	3.3 (2.9–3.8)	Ref	31	0.5 (0.4–0.7)	74	1.2 (1.0–1.5)
No fixed address	155	19	12.3 (8.0–18.4)	2.9 (1.5–5.5)	0	0	12	7.7 (4.5–13.1)
**Total (crude)^b^**	**6,211**	**217**	**3.5 (3.1–4.0)**	**NA**	**31**	**0.5 (0.4–0.7)**	**86**	**1.4 (1.1–1.7)**
**Total (adjusted)^c^**	**6,108**	**214**	**4.2 (3.6–4.9)**	**NA**	**31**	**0.7 (0.4–1.0)**	**83**	**1.6 (1.3–2.1)**

aOR: adjusted odds ratio; BBV: blood-borne virus; CI: confidence interval; NA: not applicable; Ref: reference; RLTC: requiring linkage to care.

^a^ aOR and 95% CI for the association between demographic factors and positive serology for at least one blood-borne virus (HIV, HBV and/or HCV RNA). Estimates were weighted to reflect the age, sex and ethnicity of the FBC population, after excluding those with unclassified age and sex (n = 24,321). Wald test p values: p < 0.0001 for sex and ethnicity, p = 0.0001 for age and p = 0.0017 for no fixed address.

^b^ The 217 BBV cases were unique patients including eight co-infections.

^c^ Adjusted prevalence estimates were weighted to reflect the age, sex and ethnicity of the FBC population, after excluding those with unclassified age and sex (n = 24,321).

### Diagnostic status and linkage to care outcomes among BBV cases

Over half of the 217 BBV cases were classified as known-engaged (129 cases; 59%), with much smaller proportions of known-disengaged (32 cases; 15%) and new diagnoses (31 cases; 14%). The remaining 25 cases (12%) were either uncontactable or known-unknown.

Among the 86 cases RLTC (40% of the 217 BBV cases), 58 (67%) were notified of their diagnosis, 47 (55%) were partially linked to care and 35 (41%) were fully linked to care. BBV cases with no fixed address were few in number (19/217 cases), but were disproportionately hard to follow-up, with over half (11 cases) remaining uncontactable and only one notified of their test result (Figure).

### Numbers needed to screen

NNS to detect a new BBV diagnosis was 154 (95% CI: 103–233) and to fully link a BBV case to care was 135 (95% CI: 93–200).

### Summary of infection-specific results

#### Seroprevalence and factors associated with positive serology

Infection-specific adjusted seroprevalence was estimated at 2.6% (95% CI: 2.2–3.2; n = 147) for HCV-Ab, 1.8% (95% CI: 1.4–2.2; n = 100) for HCV RNA, 1.5% (95% CI: 1.2–2.0; n = 71) for HIV and 1.1% (95% CI: 0.8–1.5; n = 54) for HBV. There was no significant difference between the adjusted estimates of seroprevalence among new diagnoses for HCV RNA (0.3%; 95% CI: 0.2–0.5; n = 13), HBV (0.1%; 95% CI: 0.1–0.3; n = 8) and HIV (0.3%; 95% CI: 0.1–0.5; n = 10). Adjusted prevalence among cases RLTC was highest for HCV RNA (0.8%; 95% CI: 0.6–1.1; n = 44), followed by HBV (0.5%; 95% CI: 0.3–0.8; n = 27) then HIV (0.4%; 95% CI: 0.2–0.6; n = 16).

Being male, 40–59 years old or having no fixed address were all associated with increased adjusted odds of positive HCV-RNA serology. For HIV, males were more likely and older patients (60–89 years) were less likely to be positive. There was no association between positive HBV serology and age, sex or having a fixed address, in the multivariable analysis. Associations with ethnicity varied by infection; the Asian British/Asian other group was less likely to be positive for both HCV RNA and HIV, whereas the Black British/Black other group was associated with reduced odds of HCV RNA, but increased odds of positive HIV serology. There was considerable uncertainty in the estimates for HBV due to small counts in the White British reference category, but both the crude seroprevalence estimates and the aOR from the multivariable analysis pointed to elevated odds of positive HBV serology among the White other, Black British/Black other, Asian British/Asian other and Mixed or other ethnic groups (Supplementary Tables S1, S2 and S3).

#### Diagnostic status and linkage to care

For each infection, the highest proportion of cases were classified as known-engaged, ranging from 48% for HBV to 77% for HIV. For the three infections, 13–15% of cases were new diagnoses. The proportion of cases RLTC was lowest for HIV (23%; 16/71 cases), followed by HCV RNA (44%; 44/100 cases) and HBV (50%; 27/54 cases). HIV cases remained unlinked less often than the others (4/16 RLTC compared with 10/27 HBV and 14/44 HCV) and had the highest proportion of fully linked cases (69%), though the total number of fully linked cases for each infection were very similar (Figure).

#### Numbers needed to screen

NNS to detect a new diagnosis were 400 (95% CI: 204–769) for HIV, 833 (95% CI: 370–1,667) for HBV and 357 (95% CI: 185–667) for HCV RNA, and to fully link a case to care were 385 (95% CI: 208–714) for HIV, 588 (95% CI: 294–1,111) for HBV and 313 (95% CI: 167–588) for HCV RNA.

## Discussion

This programme resulted in the identification of 217 BBV cases from 6,211 patients tested for at least two of the three selected BBVs, giving an adjusted estimate of overall BBV seroprevalence among patients who had a FBC of 4.2% (95% CI: 3.6–4.9). Elevated aOR of positive BBV serology were estimated in FBC patients that were male (2.7; 95% CI: 1.9–3.9), 40–59 years old (1.9; 95% CI: 1.4–2.7), of Black British/Black other ethnicity (1.8; 95% CI: 1.2–2.8) or who had no fixed address (2.9; 95% CI: 1.5–5.5). We estimated that 154 (95% CI: 103–233) FBC patients needed to be screened to detect one new BBV diagnosis and 135 (95% CI: 93–200) needed to be screened to detect a BBV case that was fully linked to care.

The implementation of this programme as part of normal workflows in an ED introduced a number of limitations. Only routinely collected demographic information was recorded, which meant that we were unable to account for known risk factors, such as those of people who inject drugs (PWID) [3,10,11] or men who have sex with men (MSM) [3,9]. Originating from a country where BBVs are more prevalent [3,29-32] has been postulated as a predictor of infection rates similar to those in the country of origin [11]. Self-reported ethnicity, as collected in this programme, may be a less reliable determinant of disease risk than country of birth and time spent in the UK [33,34]—neither of which were recorded in the ED. Further, small numbers of cases, particularly for HBV, may have led to missed associations between demographic factors and positive disease serology due to low statistical power. In addition, patients could only be recruited for testing via convenience sampling; such non-probability sampling is prone to selection bias and the differential BBV testing uptake that we identified among certain groups, as reported elsewhere [17,19], may be evidence of this. Unlike in other studies [19], reasons for not testing were not recorded; relevant factors might include patients’ refusal, a previous diagnosis, inconsistent offering of testing or targeting of particular groups, all of which could bias our estimates in different ways. However, the use of weighting to adjust for non-participation in testing aimed to address this and is an improvement over previous ED-based programmes that have only reported crude prevalence [17,18,23-25].

The demographic factors we identified as associated with positive BBV serology were broadly in line with those reported previously for London and England [3,4,8-11,28,34]. One exception is our finding of Asian British/Asian other ethnicity (which was dominated by Bangladeshis, followed by ‘other Asian’, Indians, Pakistanis and a small number of Chinese) being strongly protective against positive HCV serology; this runs counter to modelled estimates that show HCV prevalence among those who are not PWID in London to be much higher in people of south Asian ethnicity than all other ethnic groups [11]. Unmeasured MSM status may partly explain the elevated odds of HIV we detected among males, since HIV prevalence among MSM in London (13.5%) is estimated to be around four times higher than in the rest of England and Wales (3.9%) [14]. There was less clear agreement between our seroprevalence estimates and published estimates of prevalence in those aged 15–59 years in London for HCV-Ab (1.2%) [11] and diagnosed HIV (0.6%) [12]. We obtained much higher estimates for our population who had a FBC and were aged 18–59 years for HIV (1.9%; 1.5–2.5) and HCV-Ab (2.8%; 2.3–3.4). Our elevated prevalence estimates may by partly due to a genuinely higher underlying HCV and HIV prevalence in the area served by the hospital, as compared to the rest of London; however, it is also highly unlikely that ED attendees, and the subset of them that require a FBC, represent the general population of either the hospital’s catchment area or the rest of London, in terms of risk factors for BBVs. With the exception of HCV-Ab prevalence estimated from an ED in Dublin (5.1%; 95% CI: 4.6–5.5) that was more than twice as high as we observed [23], there was, strong agreement between our crude estimates of infection-specific seroprevalence and those reported from previous urban ED testing campaigns in London (all BBVs) [17,18,35], Dublin (HIV and HBV) [23], Germany (HCV-Ab) [25], Switzerland (HCV-Ab) [24] and the Netherlands (HIV) [36].

Numerous lessons for ED-based testing programmes have emerged from this work. Our low testing uptake might be improved by investigating and adopting the methods used in hospitals that previously reported higher rates [17,19,23]; one promising approach is a pre-selected test-ordering IT system used in a recent ED-based programme in London, which reported 56% testing uptake [35]. A major limitation of our programme was that over half of the BBV cases we identified were already engaged in care; detection of these is useful for understanding seroprevalence, but is wasteful if the objective of a programme is to find cases RLTC, which is where the main public health return on investment (ROI) lies. Approaches to exclude known-engaged patients could include asking about recent testing [37] or the use of an automatic hospital record check, although the feasibility of either approach in a busy ED would need to be assessed and some patients may not always choose to disclose their status and be tested anyway. Targeted testing of groups known to be at the highest risk of BBV infection, including those identified in this study, may also improve the ROI of future programmes, though it would be advisable to evaluate the sensitivity of targeted vs universal screening [22] and to first decide what is an acceptable level of missed diagnoses. Cases with no fixed address were particularly hard to contact and therefore link to care. LTC rates might therefore be improved through the use of community settings, such as homeless outreach services or needle exchange centres, to contact and inform those who are homeless or currently injecting drugs of their diagnoses, carry out clinical assessments and discuss treatment options.

### Conclusion

In conclusion, the low NNS estimated by this programme suggest routine BBV screening in the ED may be worthwhile, though a formal cost-effectiveness appraisal, which was beyond the scope of this work, is advisable. We identified high BBV seroprevalence, in line with previously reported estimates from similar programmes in Western Europe. Our adjusted estimates of seroprevalence and demographic factors associated with positive BBV serology are likely to be generalisable to patients having a FBC in EDs in London or other large UK cities, and may be relevant to EDs in Western European cities with BBV epidemiology similar to London’s. Those considering similar programmes should use our findings to inform their assessments of the anticipated public health benefits of the intervention and in the planning of its delivery.

## Supplementary Data

247000 Supplementary Tables S1
