# Supplementary material for: Seroprevalence and demographic factors associated with hepatitis B, hepatitis C and HIV infection from a hospital emergency department testing programme, London, United Kingdom, 2015 to 2016
Source: Euro Surveill. 2019 Jul 4;24(27):1800377. doi: 10.2807/1560-7917.ES.2019.24.27.1800377 (PMC6628754; doi:10.2807/1560-7917.ES.2019.24.27.1800377)
Supplement: Supplementary Tables S1 [file 1800377_BUNDLE_SupplementaryTables.pdf]

Supplementary materials for Seroprevalence and demographic factors associated with hepatitis B, hepatitis C and HIV infection from a hospital emergency department testing programme, London, United Kingdom, 2015 to 2016

Disclaimer: This supplementary material is hosted by *Eurosurveillance* as supporting information alongside the article *Seroprevalence and demographic factors associated with hepatitis B, hepatitis C and HIV infection from a hospital emergency department testing programme, London, United Kingdom, 2015 to 2016* on behalf of the authors who remain responsible for the accuracy and appropriateness of the content. The same standards for ethics, copyright, attributions and permissions as for the article apply. *Eurosurveillance* is not responsible for the maintenance of any links or email addresses provided therein.

Table S1: Seroprevalence of HIV and demographic factors associated with positive HIV serology among patients having a full blood count, hospital emergency department testing programme, London, United Kingdom, 2015–2016 (n=71)

Characteristic	Number tested	All cases			New diagnoses		Requiring linkage	
		n	Prevalence % (95% CI)	aOR (95% CI)[a]	n	Prevalence % (95% CI)	n	Prevalence % (95% CI)
**Sex**								
Female	2,852	14	0.5 (0.3-0.8)	(ref)	2	0.1 (0.0-0.3)	3	0.1 (0.0-0.3)
Male	3,240	57	1.8 (1.4-2.3)	3.1 (1.7-5.7)	8	0.3 (0.1-0.5)	13	0.4 (0.2-0.7)
**Age (years)**								
18-39	1,546	30	1.1 (0.7-1.5)	(ref)	2	0.1 (0.0-0.3)	4	0.1 (0.1-0.4)
40-59	1,288	37	2.0 (1.5-2.8)	1.4 (0.86-2.5)	7	0.4 (0.2-0.8)	11	0.6 (0.3-1.1)
60-89	1,028	4	0.3 (0.1-0.8)	0.30 (0.10-0.95)	1	0.1 (0.0-0.5)	1	0.1 (0.0-0.5)
Unclassified	102	0	0	n/a	0	0	0	0
**Ethnicity**								
White British	1,676	15	0.9 (0.5-1.5)	(ref)	1	0.1 (0.0-0.4)	2	0.1 (0.0-0.5)
White other (incl. Irish)	797	12	1.5 (0.9-2.6)	1.5 (0.86-2.5)	3	0.4 (0.1-1.2)	4	0.5 (0.2-1.3)
Asian British or Asian other	1,730	3	0.2 (0.1-0.5)	0.20 (0.06-0.71)	1	0.1 (0.0-0.4)	2	0.1 (0.0-0.5)
Black British or Black other	593	26	4.4 (3.0-6.4)	4.8 (2.4-9.1)	4	0.7 (0.3-1.8)	5	0.8 (0.4-2.0)
Mixed or other	607	7	1.2 (0.6-2.4)	1.1 (0.43-2.8)	0	0	1	0.2 (0.0-1.2)
Ethnicity not recorded	689	8	1.2 (0.6-2.3)	1.4 (0.56-3.5)	1	0.2 (0.0-1.0)	2	0.3 (0.1-1.2)
**Residence**								
Fixed address	5,938	70	1.2 (0.9-1.5)		10	0.2 (0.1-0.3)	15	0.3 (0.2-0.4)
No fixed address	154	1	0.7 (0.1-4.5)		0	0	1	0.7 (0.1-4.5)
Total (crude)	6,092	71	1.2 (0.9-1.5)	n/a	10	0.2 (0.1-0.3)	16	0.3 (0.2-0.4)
Total (adjusted)[b]	5,990	71	1.5 (1.2-2.0)	n/a	10	0.3 (0.1-0.5)	16	0.4 (0.2-0.6)

[a] Adjusted odds ratio (aOR) and 95% confidence interval (CI) for the association between demographic factors and positive HIV serology. Estimates were weighted to reflect the age, sex and ethnicity of the FBC population having excluded observations with unclassified age and sex (n=24,321). Wald test p-values: p=0.0003 for sex, p=0.0185 for age and p<0.0001 for ethnicity. No fixed address was not included in the model as it was not significantly associated with positivity in single variable analysis (p=0.546)

[b] Adjusted prevalence estimates weighted to reflect the age, sex and ethnicity of the FBC population, after excluding those with unclassified age and sex (n=24,321)

Table S2: Seroprevalence of HBV and demographic factors associated with positive HBV serology among patients having a full blood count, hospital emergency department testing programme, London, United Kingdom, 2015–2016 (n=54)

Characteristic	Number tested	All cases			New diagnoses		Requiring linkage	
		n	Prevalence % (95% CI)	aOR (95% CI)[a]	n	Prevalence % (95% CI)	n	Prevalence % (95% CI)
**Sex**								
Female	2,843	18	0.6 (0.4-1.0)	(ref)	2	0.1 (0.0-0.3)	5	0.2 (0.1-0.4)
Male	3,180	36	1.1 (0.8-1.6)	1.7 (0.88-3.2)	6	0.2 (0.1-0.4)	22	0.7 (0.5-1.1)
**Age (years)**								
18-39	2,755	18	0.7 (0.4-1.0)	(ref)	5	0.2 (0.1-0.4)	9	0.3 (0.2-0.6)
40-59	1,833	22	1.0 (0.8-1.8)	1.8 (0.90-3.5)	3	0.2 (0.1-0.5)	12	0.7 (0.4-1.2)
60-89	1,334	11	0.8 (0.5-1.5)	1.5 (0.66-3.5)	0	0	3	0.2 (0.1-0.7)
Unclassified	101	3	3.0 (1.0-8.8)	n/a	0	0	3	3.0 (1.0-8.8)
**Ethnicity**								
White British	1,651	2	0.1 (0.0-0.5)	(ref)	1	0.1 (0.0-0.4)	2	0.1 (0.0-0.5)
White other (incl. Irish)	787	12	1.5 (0.9-2.7)	15 (3.3-71)	2	0.3 (0.1-1.0)	8	1.0 (0.5-2.0)
Asian British or Asian other	1,707	15	0.9 (0.5-1.5)	8.1 (1.8-36)	2	0.1 (0.0-0.5)	5	0.3 (0.1-0.7)
Black British or Black other	589	14	2.4 (1.4-4.0)	20 (4.5-91)	0	0	2	0.3 (0.1-1.4)
Mixed or other	597	5	0.8 (0.3-2.0)	6.3 (1.2-34)	2	0.3 (0.1-1.3)	4	0.7 (0.3-1.8)
Ethnicity not recorded	692	6	0.9 (0.4-1.9)	4.4 (0.69-29)	1	0.1 (0.0-1.0)	6	0.9 (0.4-1.9)
**Residence**								
Fixed address	5,869	50	0.9 (0.7-1.1)	(ref)	8	0.1 (0.1-0.3)	23	0.4 (0.3-0.6)
No fixed address	154	4	2.6 (1.0-6.7)	1.3 (0.17-9.5)	0	0	4	2.6 (1.0-6.7)
Total (crude)	6,023	54	0.9 (0.7-1.2)	n/a	8	0.1 (0.1-0.3)	27	0.5 (0.3-0.7)
Total (adjusted)[b]	5,922	51	1.1 (0.8-1.5)	n/a	8	0.1 (0.1-0.3)	24	0.5 (0.3-0.8)

[a] Adjusted odds ratio (aOR) and 95% confidence interval (CI) for the association between demographic factors and positive HBV serology. Estimates were weighted to reflect the age, sex and ethnicity of the FBC population having excluded observations with unclassified age and sex (n=24,321). Wald test p-values: p=0.1150 for sex, p=0.2447 for age, p=0.0007 for ethnicity, p=0.8156 for no fixed address

[b] Adjusted prevalence estimates weighted to reflect the age, sex and ethnicity of the FBC population, after excluding those with unclassified age and sex (n=24,321)

Table S3: Seroprevalence of HCV-Ab, HCV-RNA and demographic factors associated with positive HCV-RNA serology among patients having a full blood count, hospital emergency department testing programme, London, United Kingdom, 2015–2016 (n=147)

		HCV-Ab		HCV-RNA						
		All cases		All cases			New diagnoses		Requiring linkage	
**Characteristic**	**Number tested**	**n**	**Prevalence % (95% CI)**	**n**	**Prevalence % (95% CI)**	**aOR (95% CI)[a]**	**n**	**Prevalence % (95% CI)**	**n**	**Prevalence % (95% CI)**
**Sex**										
Female	2,877	42	1.5 (1.1-2.0)	21	0.7 (0.5-1.1)	(ref)	4	0.1 (0.1-0.4)	13	0.5 (0.3-0.8)
Male	3,282	105	3.2 (2.7-3.9)	79	2.4 (1.9-3.0)	3.0 (1.7-5.5)	9	0.3 (0.1-0.5)	31	0.9 (0.7-1.3)
**Age (years)**										
18-39	2,848	42	1.5 (1.1-2.0)	32	1.1 (0.8-1.6)	(ref)	4	0.1 (0.1-0.4)	16	0.6 (0.3-1.0)
40-59	1,856	83	4.5 (3.6-5.5)	56	3.0 (2.3-3.9)	2.6 (1.6-4.2)	6	0.3 (0.2-0.7)	23	1.2 (0.8-1.9)
60-89	1,352	21	1.6 (1.0-2.4)	12	0.9 (0.5-1.6)	1.4 (0.64-3.1)	3	0.2 (0.1-0.7)	5	0.4 (0.2-0.9)
Unclassified	103	1	1.0 (0.1-6.6)	0	0	n/a	0	0	0	0
**Ethnicity**										
White British	1,697	73	4.3 (3.4-5.4)	50	3.0 (2.2-3.9)	(ref)	4	0.2 (0.1-0.6)	19	1.1 (0.7-1.8)
White other (incl. Irish)	817	31	3.8 (2.7-5.4)	20	2.5 (1.6-3.8)	0.94 (0.52-1.7)	5	0.6 (0.3-1.5)	13	1.6 (0.9-2.7)
Asian British or Asian other	1,733	8	0.5 (0.2-0.9)	5	0.3 (0.1-0.7)	0.10 (0.04-0.26)	1	0.1 (0.0-0.4)	2	0.1 (0.0-0.5)
Black British or Black other	595	7	1.2 (0.5-2.5)	5	0.8 (0.4-2.0)	0.29 (0.11-0.75)	1	0.2 (0.0-1.2)	3	0.5 (0.2-1.6)
Mixed or other	609	17	2.8 (1.7-4.4)	15	2.5 (1.5-4.1)	0.91 (0.46-1.8)	2	0.3 (0.1-1.3)	6	1.0 (0.4-2.2)
Ethnicity not recorded	708	11	1.6 (0.9-2.8)	5	0.7 (0.3-1.7)	0.18 (0.06-0.53)	0	0	1	0.1 (0.0-1.0)
**Residence**										
Fixed address	6,005	130	2.1 (1.8-2.6)	86	1.4 (1.2-1.8)	(ref)	13	0.2 (0.1-0.4)	37	0.6 (0.5-0.9)
No fixed address	154	17	11.0 (7.0-17.0)	14	9.1 (5.5-14.8)	5.8 (2.7-12)	0	0	7	4.6 (2.2-9.2)
Total (crude)	6,159	147	2.4 (2.0-2.8)	100	1.6 (1.3-2.0)	n/a	13	0.2 (0.1-0.4)	44	0.7 (0.5-1.0)
Total (adjusted)[b]	6,056	146	2.6 (2.2-3.2)	100	1.8 (1.4-2.2)	n/a	13	0.3 (0.2-0.5)	44	0.8 (0.6-1.1)

[a] Adjusted odds ratio (aOR) and 95% confidence interval (CI) for the association between demographic factors and positive HCV-RNA serology. Estimates were weighted to reflect the age, sex and ethnicity of the FBC population having excluded observations with unclassified age and sex (n=24,321). Wald test p-values: p=0.0002 for sex and age, p<0.0001 for ethnicity and no fixed address

[b] Adjusted prevalence estimates weighted to reflect the age, sex and ethnicity of the FBC population having excluded observations with unclassified age and sex (n=24,321)
